# The deployment of social media by political authorities and health experts to enhance public information during the COVID-19 pandemic

**DOI:** 10.1016/j.ssmph.2022.101165

**Published:** 2022-07-08

**Authors:** Maud Reveilhac

**Affiliations:** Lausanne University, Faculty of Social and Political Sciences, Institute of Social Sciences, Life Course and Social Inequality Research Centre, Switzerland

**Keywords:** COVID-19, Social media, Public authorities, Content analysis, Public trust

## Abstract

Social media have increasingly been used by political bodies and experts to disseminate health information to the public. However, we still know little about how the communication of these actors on social media is received by other users and how it reflects trends in public trust. We examined social media dynamics in the communication of information by major actors (n = 188) involved in COVID-19 online discussions in Switzerland. These actors are scientists (experts), policymakers (government officials, cantonal executives, and other parties), and representatives of mass media. We found little correlation between Twitter features (other users' engagement and negativity in other users' replies) and the level of public trust found in representative opinion surveys. We used topic modelling in combination with correspondence analysis, and including additional variables for actor types and the period of the public debate further enabled us to detect salient episodes related to the pandemic on social media. In particular, we found that differing roles were played by the (health) experts and political authorities in terms of both topics and influence on the specific timing of the pandemic. The results of this study provide helpful conclusions for communication among political authorities, health experts, and the public.

## Highlights

• Analysis of two years of tweets by policy makers and health experts about Covid-19.
• Correlation between tweet reception by other users and public trust.
• Extraction of salient episodes of the pandemic with correspondence analysis.
• Guidelines for improving communication with the population on social media.

## 1 Introduction: Political authorities' and experts' use of social media to inform the public about health issues

Social media have increasingly been used by officials – such as political authorities and health experts – to disseminate health information to the public (Gough et al., 2017). This trend reached unprecedented levels during the COVID-19 pandemic in efforts to enhance trust in scientific expertise (van Dijck & Alinejad, 2020). In parallel, there was an increase in citizens' reliance on social media to obtain news and COVID-19-related information (Nielsen et al., 2020). Social media platforms thus played an important role in political authorities' communication with citizens as the pandemic led to a narrowing of the topic agenda on these platforms, with an increased level of Twitter activity by political and health experts (Rauchfleisch, Vogler & Eisenegger, 2021). Specific health and political actors' communications are also likely to be directed to (or at least mention) target population groups, whose acceptance of the measures is essential to achieve the desired policy outcome (e.g., Martin et al., 2020).

Nevertheless, the roles of social media and public trust during the COVID-19 pandemic are relatively new topics that deserve more attention from the research community. Researchers still possess little knowledge of how public trust in health experts and political authorities is comparable with social media trends during an epidemic. Our study contributes to understanding these processes by drawing on the Swiss context. We are interested in answering the following research questions: Is the level of public trust measured through opinion surveys also reflected in social media users' engagement with messages from figures of authority? What were salient clusters of the COVID-19-related online discussions? How were these clusters received by the broader audience?

The investigation of levels of public trust in (political and health) authorities in the context of (health) crises is not new. Most such studies have relied on survey data. For instance, psychological variables, such as trust and worldview, strongly influence people's risk perceptions and acceptance of health measures (Siegrist & Bearth, 2021). Sustaining trust has thus been a challenge over the course of the COVID-19 pandemic. Previous studies have found that trust in health authorities and government institutions is importantly correlated with citizens' compliance with public health policies and guidelines (Blair et al., 2017; Vinck et al., 2019). Public trust in political authorities can also be influenced by social media. Indeed, previous studies have identified criticisms of political authorities and institutions, thus suggesting an expression of distrust in political authorities (Linde-Arias et al., 2020; Roy et al., 2020).

In addition to survey data, social media have offered important advantages in delivering interactive communications between political authorities and citizens during the COVID-19 pandemic (Chen et al., 2020). Furthermore, social media are a useful source of data for rapid and exhaustive data collection to support evidence-based decisions based on public reactions (Huerta et al., 2021) when traditional face-to-face approaches are deemed difficult (Grow et al., 2020). Nonetheless, this focus on social media communication does not come without challenges, as these platforms also constitute a means for citizens to bypass traditional media outlets, provide channels for carrying out verbal attacks on political authorities and facilitate the spread of mis/disinformation (Brennen et al., 2020).

Empirical evidence on how trust in health experts and political authorities has evolved in the different phases of the COVID-19 pandemic, acquired by comparing offline and online trends, is needed. Furthermore, van Dijck and Alinejad (2020) found evidence that the current phase of the public debate is an important factor that affects opportunities for actors to communicate with the public. In this regard, our study shows how the reception of the online communications of political authorities during the pandemic can provide us with complementary insights to better grasp the levels of trust measured in opinion surveys. In particular, we draw on two complementary measures of public engagement related to major actors' handling of the COVID-19 pandemic in Switzerland. These actors included not only health officials (e.g., Federal Office of Public Health (FOPH) and Taskforce experts) and political authorities (e.g., Federal Council, cantonal executives, members of Parliament, representatives of national parties, and elected politicians) but also the media, important economic actors, and universities. Including social media and survey data within the same study enables us to investigate the real-time dynamics of public engagement and trust in scientists and public health authorities.

We conducted several research steps:

First, we benchmarked how these actors' social media messages were received by the broader online audience compared to survey measures of public trust in similar actors. As a reception measure on social media, we relied on negativity in replies and on the sum of interactive features (e.g., likes and retweets) attached to messages from health and political authorities. Liking and retweeting are low-effort interactions that likely indicate endorsement and support of the original message. For this reason, they convey a measure of the popularity of a message (Guerrero-Solé, 2016), and summing these interactions provides us with a reasonable measure of engagement. In contrast to retweets and likes, interactions with replies require writing and do not necessarily reflect users' support. Therefore, we also relied on the overall negativity of user replies to messages from health and political authorities, which was assessed through dictionary-based sentiment detection.

Second, in line with previous studies demonstrating the usefulness of including the main topics of discussion among the active variables used in correspondence analysis (Gesualdo et al., 2022; Zengul et al., 2021), we identified major associations in online discussions of the COVID-19 pandemic that were characterized by different political and health authorities, by the phases of the COVID-19 pandemic, by the topics extracted through automatic text classification, and by the mention of target populations. The main clusters of discussions were then approached with hierarchical clustering to consider how they were characterized in terms of interactive features (e.g., likes and retweets) and of the negativity in other users' replies.

## 2 Study background

### 2.1 The role of social media in crisis communication

Despite the existence of studies underlining the role of social media in informing the dynamics of the public debate about the pandemic, few studies have relied on social media to examine political authorities' communication behaviour when attempting to raise public awareness (Zhao et al., 2020). Research on political authorities' use of social media to increase public attention to epidemics has been conducted in the contexts of the Ebola outbreak (Strekalova, 2017) and the H1N1 pandemic (Liu & Kim, 2011).

Shortly after its emergence, COVID-19 became one of the major concerns for policymakers and the public worldwide. Meanwhile, social media platforms have increasingly become primary sources of news and information (Mitchell, 2016). In this context, political authorities and health experts have strong incentives to maximize their social media efforts, especially during crises (Graham et al., 2015; Tagliacozzo & Magni, 2018). During the COVID-19 pandemic, social media have played a particularly important role in many countries (Sha et al., 2020; Thelwall & Thelwall, 2020), but they have also been linked to the spread of misinformation (Bauer, Freitag, & Sciarini, 2013).

Studies using social media to measure these attitudes and behaviours in relation to actors' communication have found that actor expertise has an important impact, as users' exposure to scientific social media messages leads to improved public knowledge (Vraga & Bode, 2017). Furthermore, evidence has suggested that mass publics are receptive to important information from governments (Goldberg et al., 2020). Both political communication and scientific expertise converge in their intention to incentivize specific behaviour and attitude changes. Indeed, government communication during crises is most effective when it translates scientific and technical information (Herovic et al., 2020). Gilardi, Gessler, Kubli, and Müller (2021) further found that social media challenge the capacity of party and media elites to craft a consensus regarding the appropriateness of different measures as responses to COVID-19.

However, social media also challenge political authorities' communication, as they allow multiple stakeholders and groups to shape social and political agendas while bypassing traditional gatekeepers such as news media (Jungherr & Gayo-Avello, 2020). For instance, Gilardi et al. (2021) investigated policy responses to COVID-19 promoted by Swiss political and health authorities, with a special focus on policy solutions, namely, face mask rules and contact tracing apps. The authors analysed the salience of these policy solutions to the COVID-19 problem. They found that the debate on face masks was led by the attentive public (a group of users who follow the accounts of at least five Swiss news outlets) and by politicians, followed by parties and newspapers. Social media thus challenge the capacity of party and media elites to elaborate appropriate measures as responses to a major health crisis.

In addition to the type of actors engaged in this debate, the stage that the public debate has reached is an important factor influencing how actors' messages are received by the public. The development of crisis management strategies by actors can indeed highly impact perceptions of their institutional expertise for handling the pandemic. For instance, van Dijck and Alinejad (2020) showed that the phases in the public debate were an important factor affecting how institutional actors engaged in communication with the public on social media. Social media are deployed to both undermine and enhance public trust in scientific expertise, and actors need to adapt their communication at the various stages of a public debate.

### 2.2 Measuring the success of actors' messages on social media

In public health crises, the communication of actors, such as political elites and health experts, is crucial for compliance with policy measures. When communicating with the public, these actors aim to reach target audiences with a view of inducing behavioural and attitudinal changes (Vinck et al., 2019). Measuring these changes is extremely difficult, but social media provide us with strong signals of how actors' messages are received by the public. The main way of interpreting these data consists of noting reactions (Cho et al., 2014) – such as expressions of satisfaction (e.g., likes), responses to messages (e.g., replies), and the propagation of messages (e.g., retweets) – which are widely used metrics in social media research (Stone & Can, 2020).

Against this background, it is important to measure the success of actors' messages, in particular by assessing the dynamics of the debate between actors and citizens but also by measuring the extent to which citizens spread these messages and interact with them. However, users choosing to engage in online debate about the COVID-19 pandemic may not be representative of the average person in terms of behavioural characteristics. For instance, social media discussions can be ideologically polarized and organized in echo chambers, as has been demonstrated in the case of the vaccination debate in Italy (Cossard et al., 2020) or of COVID-19 discussions in the United States (Jiang et al., 2020). Even though social media users are generally unrepresentative of the general public (Mellon & Prosser, 2017), studying social media reactions towards political authorities can still validly provide us with complementary information about the public attitudinal dynamics towards political authorities and health experts.

Several studies have investigated how political authorities' communication is received by a broader public of social media users. For instance, Raamkumar, Tan, and Wee (2020) examined the COVID-19-related outreach efforts of public health institutions in Singapore, the United States, and England and the corresponding public responses to these outreach efforts on Facebook. Using sentiment analysis, the authors found cross-country variations between overall sentiments towards public health authorities. Moreover, Mahdikhani (2021) studied public opinion and emotions at different stages of the COVID-19 pandemic, from the outbreak of the disease to the distribution of vaccines, and found that tweets with higher emotional intensity were more popular than tweets containing information about the COVID-19 pandemic. Furthermore, Teichmann et al. (2020) showed that different posting strategies on Twitter and Facebook were effective in drawing public attention to political authorities' health messages. For instance, the poster could often be more important than what was posted, and concise messages with clearly formulated health directives tended to receive widespread engagement. However, evidence suggested that health authorities faced low engagement with their social media posts related to the pandemic (Berg et al., 2021).

### 2.3 Comparing survey measures of public trust with social media reactions

To date, the most efficient way of accounting for public trust in political authorities and for public approval of policy measures is to rely on opinion surveys. In the context of COVID-19, national and international surveys have been developed to understand how public attitudes and behaviours are evolving in relation to the pandemic. Contrary to surveys that pose questions on well-defined concepts but usually require intensive resources to collect data, social media provide signals of opinions that can be accessed in a timely manner without the intervention of researchers (Diaz et al., 2016).

However, social media data are often messy, thus requiring specific pretreatments and cleanings before they can be meaningfully analysed (Klašnja et al., 2015). They are also usually not representative of national populations and lack information about personal attributes (e.g., Barbera & Rivero, 2014). Indeed, social media tend to be used predominantly by active user groups – such as politicians, influencers, journalists, and bots – who are influential in terms of public opinion (e.g., Hargittai, 2018), while survey data aim to be representative of what the wider public thinks.

Due to their respective characteristics, comparing the two data sources can provide meaningful insights into the congruence between offline and online support of political authorities and the policy measures that they propose to fight the COVID-19 pandemic. We view this comparison as useful for informing future actors' reliance on social media as a means of communicating with the public on health issues. More specifically, it enables us to assess whether their perceived trustworthiness within the population is congruent with how their communications are received online.

### 2.4 Case study: Public trust and the stages of the COVID-19 public debate in Switzerland

People in Switzerland have demonstrated an exceptionally high level of confidence in their government in recent decades (Mabillard & Pasquier, 2015). This can be explained by the sense of participation in political decision-making due to (semi)direct democracy and by the trust in political authorities' communications during critical situations (Freitag & Ackermann, 2016). Furthermore, Swiss citizens are highly satisfied with their healthcare system (OECD, 2019). The Swiss population continues to rely more heavily on traditional news media than on social media for information (Reveilhac and Morselli, 2020). Humprecht et al. (2020) further found that Swiss people are more reluctant than citizens of other countries to share false information about COVID-19 on social media.

The COVID-19 pandemic has been spreading in Switzerland since February 25, 2020, the date of the first confirmed case. Each phase of the public debate has been marked by political decisions leading to protective measures being taken against the pandemic (for a chronology of the pandemic in Switzerland, see Annex 1). While a large majority of the Swiss population had confidence in the federal authorities in the first wave of the pandemic, the study conducted by Hermann et al. (2022) demonstrated a drop in confidence in the actions of governmental bodies around January 2021. This decline in the level of trust was renewed during autumn 2021, which points to the enduring polarization of society between sceptics – who show little support for measures promoted by federal institutions and generally distrust mainstream information – and people supporting the actions of governmental bodies and showing confidence in the information coming from these official bodies. The authors also found that 9% of adults in Switzerland had taken part in at least one demonstration against COVID-19 measures in the past two years of the pandemic. This number is still significantly lower than the 40% of people who voted against the two COVID-19 proposals (in June 2021 and November 2021). This climate of distrust is particularly problematic in conjunction with direct democracy systems, as it can open the door to easy populist loops (Reveilhac & Morselli, 2022).

## 3 Data and analytical strategy

### 3.1 Assessing the relationship between public trust and social media reactions

**Fig. 1:**
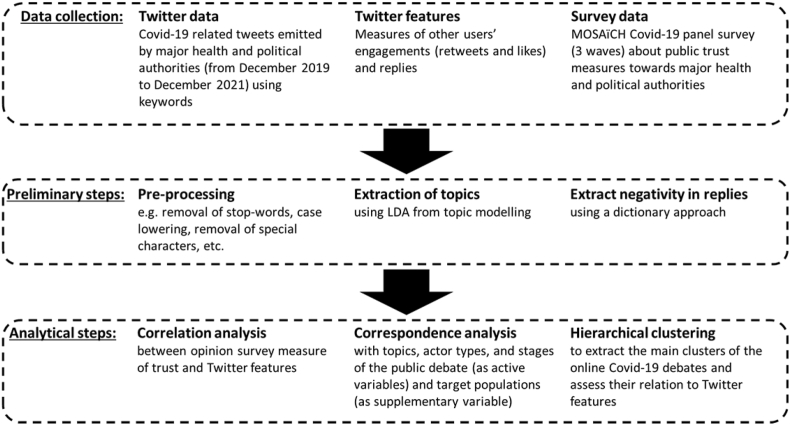
Analytical framework of the study.

To answer our first research question about whether the level of public trust measured through opinion surveys is reflected in social media users' engagement with messages from authority figures, we relied on two data sources. Fig. 1 summarizes the architecture of the study to visualize the steps related to the method and data collection:

We relied on tweets emitted by actors – namely, Swiss political authorities (the Federal Council and the Parliament as well as national parties and politicians), health experts (the FOPH and the national group of COVID-19 experts), news media (main daily or weekly newspapers), Swiss universities, and major business actors (business federations or trade unions) – from December 2019 to December 2021 (see Annex 2 for the list of Twitter accounts). We kept only original tweets and identified those related to the COVID-19 pandemic by relying on a dictionary approach using a list of COVID-19-specific search queries (see Annex 3). For all the tweets that were replied to by other users, we also retrieved these replies. Annex 4 displays the number of tweets that were collected for each group of actors and the number of selected tweets based on the list of search queries. The final sample contained 115,600 original tweets.

**Fig. 2:**
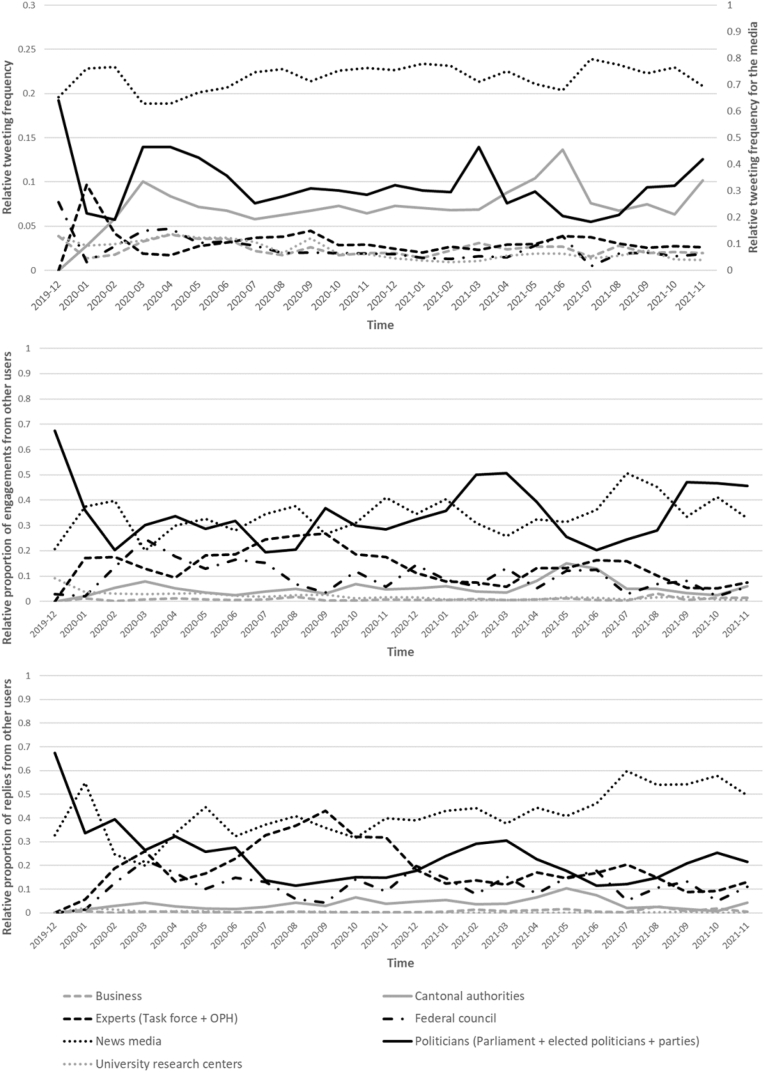
Relative share of actors' Covid-19 related tweets over time (upper left pane); Relative share of other users' reactions in terms of aggregated likes and retweets (upper right pane); Relative share of other users' replies over time (lower left pane).

Fig. 2 (upper left panel) displays the prevalence of COVID-19 tweets from the selected accounts covering the entire period under study. It is based on the relative proportion of tweets emitted on a monthly basis by each actor group (thus, the cumulative proportion for each month equals 100%). It thereby shows when each actor group was particularly active over the course of the pandemic. We see that the share of tweets was generally the most salient during the first wave of the pandemic. Peaks are also noticeable for politicians and cantonal authorities in March 2021 and June 2021 (corresponding to the dates of popular votes about the COVID-19 law).

Fig. 2 (upper right panel) shows the relative proportion of other users' reactions (likes and retweets) to each actor's tweets on a monthly basis. In general, the news media and the political elite (encompassing the Twitter accounts of the Parliament, of the elected politicians, and of the parties) trigger the largest share of other users' engagement. Furthermore, the highest share of reactions for health experts was reached before the second wave.

Fig. 2 (lower left panel) displays the relative share of replies from other users triggered by each actor group. The was a notable increase in the relative share of replies to the experts' tweets (including the Twitter accounts of the Taskforce and its members as well as the account of the FOPH). The relative share of replies to tweets from the political elite varies over time, with peaks around April 2020, March 2021, and October 2021.

### 3.2 Correlation between opinion survey measure of trust and social media features

To investigate whether the level of public trust measured through opinion surveys was also reflected in social media users' engagement with messages from figures of authority, we relied on public opinion data from the *MOSAiCH-ISSP COVID-19* survey^1^ to access measures of trust in similar official groups. The survey item used to measure trust was graded on a scale from 0 to 10 and read as follows: “How much trust do you personally have in the following institutions?” The survey spanned several waves, thereby allowing for responses to the same question to be obtained at different phases of the pandemic. We calculated the difference in public opinion between the first (from April 30th to July 13th^,^ 2020, thus beginning at the end of the first wave of the pandemic) and the third (from March 19th to April 18th^,^ 2021, thus corresponding to the third wave of the pandemic) survey waves for each institution (the second wave took place between October 2nd and November 2nd^,^ 2020).

To assess the relationship between offline and online trends, we compared the difference in surveyed public trust to reactions from other social media users. We relied on the difference in engagement metrics (including likes and retweets) and negativity in replies from other users between the periods corresponding to the first and third survey waves.

The negativity in replies was assessed for each language separately (only for German and French replies) using a dictionary-based approach. More specifically, we triangulated the *LIWC* (Pennebaker et al., 2015) and *NRC* dictionaries (Mohammad & Turney, 2013) to detect the negativity in replies from other users. Both dictionaries have been carefully translated by psychology and computational research teams in multiple languages. We label a tweet as negative if it was matched to words with a negative tonality in either dictionary.

This dictionary-based approach was chosen for this study rather than a machine learning approach. With a machine learning approach, the results would have been dependent on the quality of the training data, which, in our study, would have been difficult to obtain given that the data are in multiple languages and that human evaluators may not always be able to label them consistently according to the sentiment categories. However, to ensure that the results could identify negativity in replies with acceptable accuracy, we manually annotated a random sample of 100 replies in German and 100 replies in French. Both languages showed acceptable levels of accuracy for detecting negativity (89% in French and 92% in German). Cases where the dictionaries could not correctly identify negativity mostly contained colloquial slang or profanities. We used the negativity proportion for each actor to compute the correlation between survey and social media trends.

### 3.3 Salient topics in social media discussions

To answer our second research question about the salient clusters of the online COVID-19-related discussions and what reactions they triggered from the broader audience, we relied on two classification methods: topic modelling and correspondence analysis.

We used an unsupervised text classification method to extract relevant topics from political authorities' tweets. A “topic” consists of a cluster of words that frequently occur together and thus have similar meanings (Bauer, Freitag, & Sciarini, 2013). Documents and words were given, and topics were fitted iteratively starting from a random configuration. We used the popular implementation algorithm *latent Dirichlet allocation* (LDA), as implemented in *Mallet* software (McCallum, 2002), using a hyperparameter optimization of every 10 iterations. We preprocessed the tweets in the following sequence: i) removal of French and German stop words, ii) removal of URLs, iii) removal of special characters (e.g., *#, @*) and punctuations, iv) division of concatenated expressions (e.g., *StayAtHome* become *stay at home*), v) removal of words smaller than 2 characters, and vi) removal of words with a direct reference to COVID-19 (e.g., *covid, covid19, cov19, corona, coronavirus, pandemic*).

Topic modelling combines document classification with the strong semantic unity of the discourse of a topic and of a document by optimizing the following equation: p(topic|document) *p(word|topic), for all given documents in a collection. Document classification can be expressed by the first part of the equation: *p(topic|document)*. The task of document classification is to find the most likely class given the document (or the tweet). The second part of the above formula is the keyword generation probability: *p(word|topic).* It expresses that for a given topic, certain keywords are particularly likely.

Documents and words are given, and topics are fitted iteratively. The user must set the number of topics that the algorithm will use. This fitting process ensures that the overall probability of the given documents and words is as high as possible. We calculated the best number of topics to extract (see Annex 5, Annex 6) using the function *FindTopicsNumber()* from the *R* package *ldatuning* (Nikita & Nikita, 2016). The four metrics composing the function were computed by training several LDA models with the number of topics ranging from 5 to 200. The results suggest that the optimal number of topics with respect to these metrics is 60 for French and 70 for German tweets. The number of topics was also determined via manual inspection of a variety of topic sets trained using several different numbers of topics.

Each topic was represented by a list of top related keywords, which then needed to be manually labelled to propose a possible interpretation. We reduced the possible topic labels to 12 categories that were found to encompass enough to summarize the content of the tweets. For instance, many different topics referred to vaccination – such as laboratories, number of vaccinated people, patents, etc., and can be summarized under a single generic “vaccine” label. We assigned each tweet the topic with the highest prevalence (highest *gamma* value).

### 3.4 Salient clusters of social media discussions and other users' reactions towards these clusters

We used correspondence analysis to reveal the associations among the identified topics found in Twitter discussions, the actor type, the different stages of the public debate, and the target population, relying on the *FactoMineR* package for R (Husson et al., 2020). Hierarchical clustering was subsequently applied to extract the main clusters of discussions from the correspondence analysis results and investigate what were other users' reactions towards salient clusters of discussions.

Correspondence analysis can be understood as principal component analysis for categorical data (Reveilhac & Morselli, 2020) and is also used to discover structure in textual data (D'Enza & Greenacre, 2012). The variables are projected on a factorial space such that the proximity between variables indicates a higher association. To this aim, we relied on a two-dimensional space to plot the variables to assess how closely they related to each other. Correspondence analysis calculates the contributions of each variable to the inertia of each factorial axis.

The projected variables are distinguished between active and supplementary variables. The active variables are used for the determination of the two-dimensional space, while the coordinates of the supplementary variables are predicted using only the information provided by the performed multiple correspondence analysis on active variables. As active variables, we explored how the content of tweets related to the different actor categories while also taking the topic and the temporal dimension into account. As a supplementary variable, we considered which target population (including children, women, adults, elderly individuals, and patients) was mentioned in the tweets. The identification was based on lists of search queries (see Annex 7).

With respect to the different stages of the public debate, we differentiate between the COVID-19 waves (W0: first international increase in the number of cases from January to March 2020; W1: first increase in Swiss cases from March to April 2020; W2: second COVID-19 wave in Switzerland from October to December 2020; W3: third COVID-19 wave in Switzerland from January to May 2021; W4: fourth COVID-19 wave from September to mid-November 2021; W5: fifth wave from mid-November to mid-December 2021) and the normalization periods (N1: decrease in the number of cases and relaxation period from the end of April to mid-June 2020; N2: end of the state of emergency; N4: public spaces partially reopen from mid-May to August 2021; N5: establishment of national “2G” (vaccinated or cured) rules since mi-December 2021). Annex 1 provides a detailed description for each stage (the modalities starting with a *W* indicate the different COVID-19 waves, and the modalities starting with a *N* indicate the normalization periods).

The results of the correspondence analysis were then used to perform a hierarchical cluster analysis with the Ward method to classify the tweets of the corpus into salient momentums and to investigate the reactions of other users. The best number of clusters was determined visually (see Annex 8). Each cluster is analysed according to Twitter features (including engagement in terms of likes and retweets, as well as negativity in replies).

## 4 Results

The original COVID-19-related tweets that were collected using a list of curated search-queries represent 16% of the total tweets emitted by major actors from December 2019. The government posted significantly more about non-COVID-19-related issues than health experts did. With respect to measures of popularity, experts' tweets had the highest mean retweet rate, while the government triggered the highest mean like rate. Therefore, we observed that the main entities responsible for producing recommendations for handling the pandemic (FOPH and Taskforce) had the highest share of COVID-19-related tweets (more than 60%) compared to that of other actors. It is also interesting to note that, on average, the cantonal authorities tweeted more about COVID-19 than federal institutions. The cantons also triggered the most positive replies, thus suggesting that they were supported by the online audience. In general, the mean number of reactions (replies, retweets, and likes) was the highest for the main entities in charge of the COVID-19 communication, namely, the Federal Council and the FOPH, followed by political parties and politicians. The mean likes and retweets were also high for Taskforce actors.

To investigate how public trust in health experts and political authorities is comparable with social media trends during an epidemic, Table 1 displays the relationship between the average difference in public trust in political authorities measured between the first and the third waves of the MOSAïCH survey (y-axis) and the average differences in social media features covering the same period (x-axis). Based on Table 1, we can see that the majority of actors (except business industries, media, and research centres) suffered from a decline in public trust. Experts from the FOPH were especially affected by declining public confidence. When juxtaposed to the interactions on social media, we can see that there was an increased negativity in replies for experts and parliamentarians. However, there was a decline in the negativity in replies to the government and to the media, thus suggesting an opposing trend between survey trust and negativity in replies for these actors. Furthermore, the cantonal authorities also benefited from an increased positivity in the replies to their tweets. Table 1 also shows that the average number of engagements (in terms of likes and retweets) decreased for most actors, but especially for the government. However, there was an increased number of engagements towards parliamentarians and the media. In sum, the correlation between the survey and social media trends is generally low regarding the change in the negativity of other users' replies and with respect to engagement between the two waves. However, a closer look at each wave demonstrates a significant negative correlation between trust and negativity during the third wave.

**Table 1:** Relationship between the levels of public trust measured in surveys and the negativity in other users' replies and engagements (including likes and retweets).

	Wave 1:			Wave 3:			Difference between wave 3 and wave 1		
	(April 30th to July 13th, 2020)			(March 19th to April 18th, 2021)					
	Twitter		Survey	Twitter		Survey	Twitter		Survey
	negativity	engagement	trust	negativity	engagement	trust	negativity	engagement	trust
Business and industry	0.32	2.4	4.2	0.43	2.23	4.7	0.11	−0.17	0.5
Cantonal authorities	0.35	5.01	6.6	0.3	4.43	6	−0.05	−0.57	−0.6
Federal Office of Public Health	0.35	2.83	7.3	0.36	2.49	6.3	0.01	−0.35	−1
Federal Council	0.36	36.55	7.2	0.34	17.65	6.7	−0.02	−18.9	−0.5
News media	0.39	11.2	4.9	0.39	14.67	4.9	0	3.47	0
Parliament & elected politicians	0.35	10.73	6.3	0.37	13.88	5.9	0.02	3.15	−0.4
University research centres	0.38	6.5	7.6	0.25	5.51	7.6	−0.13	−0.99	0
**Pearson correlations of Twitter features with surveyed trust (p-value)**	**0.27 (0.26)**	**0.33 (0.56)**		**−0.87 (0.01)**	**0.09 (0.97)**		**0.28 (0.54)**	**0.21 (0.65)**	

**Fig. 3:**
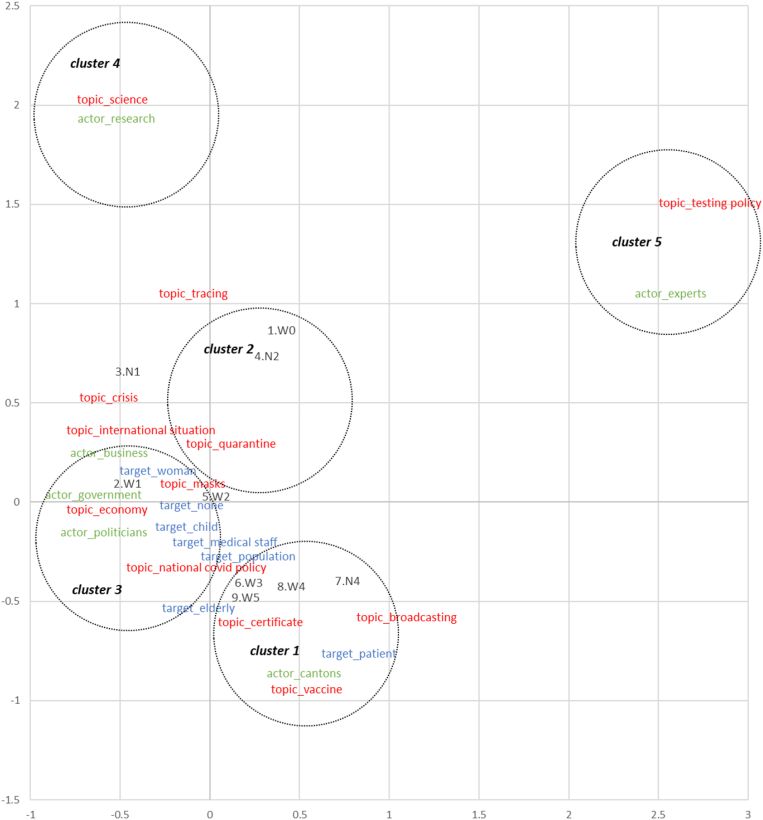
Correspondence analysis including debate stage (W indicate Covid-19 waves and N normalization periods in black), topical content (in red), actor groups (in green), and target population (in blue). (For interpretation of the references to colour in this figure legend, the reader is referred to the Web version of this article.)

Fig. 3 displays the results from the correspondence analysis along a two-dimensional space. The vertical axis is essentially useful to account for the different phases of the public debate. For instance, the first stages are represented by groups in the upper quadrants, whereas the later stages are represented by groups in the lower quadrants. The horizontal axis groups the topical content and the actor groups. Tweets from the news media were not included in the correspondence map because of their essential broadcasting behaviour and because the news media do not represent public opinion.

We can thus investigate the major accents placed by political authorities on COVID-19 by showing what topics were prioritized in their communication on social media (see also Annex 9 for the topic distribution by actor). For instance, the upper right and left quadrants show the special role of the health experts and the research centres during the pandemic. The health experts heavily promoted policy solutions (e.g., testing), while research institutes focused on technologies and tracing applications.

Furthermore, the cantons are situated apart (see lower right quadrant) from the remaining political and business actors (see centre left position on the map) and are situated close to the vaccination and certificate policies. We also see that the cantons had an increasingly important role in collecting and sharing information about new cases over the pandemic. In contrast, the government was especially active at the beginning of the pandemic and tried to inspire more confidence in COVID-19 policy measures (e.g., masks and quarantine) by accounting for the national policy while also pointing to economic difficulties and responses. The business and industry branches especially focused on the economic and international aspects of the pandemic.

Fig. 3 also enables us to assess how the major actors' communication about COVID-19 is associated with specific target populations. For instance, the cantons had a clear focus on elderly individuals and patients. Furthermore, the political and business actors focused more on women and children. References to children and to the medical staff were not significantly associated with communication.

Table 2 provides the results from the hierarchical clustering based on the previous correspondence analysis. Each cluster is characterized by tweets related to the topic content, actor groups, stages of the public debate, and target populations. In comparison to Fig. 3, additional important information provided in Annex 9 relates to the measures of Twitter features (including other users' engagement and proportion of negativity in replies) that relate to each cluster. Table 2 describes each of the seven identified clusters.

**Table 2:** Description of the clusters according to actor groups, debate stage, topical content, and target populations.

	cluster1	cluster2	cluster3	cluster4	cluster5
engagements - median	6.00	26.00	8.00	6.00	58.00
engagements - mean (sd)	27.35 (90.09)	53.63 (119.11)	37.47 (139.38)	13.8 (53.4)	71.29 (50.27)
engagements - [min. - max.]	0–2465	0–2564	0–3740	0–1695	7–509

% negativity in replies	0.32	0.36	0.36	0.32	0.35
replies – mean (sd)	5.57 (20.41)	12.00 (22.49)	4.90 (18.33)	0.70 (3.37)	30.16 (29.44)
**Stages of public debate**					
*1.W0*	0.00	0.04	0.01	0.02	0.00
*2.W1*	0.10	0.08	0.17	**0.21**	0.04
*3.N1*	0.02	0.06	**0.21**	**0.28**	0.12
*4.N2*	0.02	0.11	0.16	0.19	**0.22**
*5.W2*	0.13	0.15	0.15	0.11	0.13
*6.W3*	**0.34**	**0.26**	0.16	0.09	0.19
*7.N4*	0.19	0.16	0.06	0.05	0.16
*8.W4*	0.16	0.11	0.05	0.04	0.11
*9.W5*	0.04	0.02	0.01	0.01	0.02

**Actor groups**					
*business*	0.04	0.03	0.16	0.11	0.00
*cantonal authorities*	**0.59**	0.05	0.11	0.00	0.00
*experts*	0.00	**0.76**	0.00	0.00	**1.00**
*government*	0.06	0.04	0.14	0.01	0.00
*politicians*	**0.30**	0.11	**0.55**	0.07	0.00
*research*	0.00	0.01	0.05	**0.81**	0.00

**Topical content**					
*broadcasting*	**0.24**	**0.31**	0.03	0.00	0.00
*certificate*	0.04	0.02	0.01	0.00	0.00
*crisis*	0.01	0.02	0.13	0.14	0.00
*economy*	0.04	0.01	**0.28**	0.03	0.00
*international situation*	0.00	0.01	0.04	0.02	0.00
*masks policy*	0.03	0.02	0.09	0.06	0.00
*national covid policy*	**0.29**	0.05	**0.26**	0.07	0.00
*quarantine policy*	0.00	0.04	0.05	0.01	0.00
*science*	0.00	0.06	0.05	**0.65**	0.00
*testing policy*	0.00	**0.24**	0.00	0.00	**1.00**
*tracing*	0.00	0.03	0.03	0.03	0.00
*vaccine*	**0.35**	0.19	0.04	0.00	0.00

**Target population**					
*child*	0.04	0.04	0.04	0.06	0.00
*elderly*	0.02	0.01	0.01	0.00	0.00
*medical staff*	0.02	0.01	0.01	0.02	0.00
*patient*	0.05	0.02	0.01	0.01	0.00
*population*	0.03	0.02	0.02	0.02	0.00
*woman*	0.01	0.01	0.01	0.01	0.00
*none*	**0.84**	**0.89**	**0.91**	**0.88**	**1.00**
Number of tweets	8898	1687	9407	1559	989

Table 2 shows that cluster 1 is characteristic of the third wave of the pandemic. The cantonal authorities and politicians were particularly engaged in discussions about the national COVID-19 policy, where face masks remain an essential complementary measure to the vaccination. The level of other users' engagement is one of the lowest across the clusters (both in terms of mean and median), and the proportion of negative replies is among the lowest. However, this cluster also encompassed tweets against the national policy. For instance, the tweet that triggered the highest number of engagements was writer by a politician voicing against the enlargement of Covid-19 certificate.

Cluster 2 overlaps the third wave of the pandemic and the following normalization period. The communication of health experts (FOPH and Taskforce) is especially salient in this cluster, with a focus on testing policy and COVID-19 case broadcasting. The median number of engagements is the second highest, thus showing a high level of other users' interest and potential for supportive replies. For instance, the tweet from the group of health experts with the highest number of engagements was written by the FOPH and thanks the former head of the federal office for his devotion and years of services. However, this cluster has one of the highest proportions of negative replies, which suggests that other users were in general less supportive of the experts' communication. For instance, the tweet with the highest number of replies and the highest negativity provides an update on the quarantine obligation and the list of countries with worrisome virus variants.

Cluster 3 centres on the first normalization period of the pandemic as well as the subsequent COVID-19 waves. It especially underlines the implication of the politicians who discuss economic issues and the national COVID-19 policy. This cluster is characterized by a high share of negativity in replies and has the highest standard deviation of engagement. The tweet with the highest number of engagements was written by a politician contesting the efficiency of lockdown measures.

Cluster 4 characterizes the first wave of the pandemic and the following normalization period where researchers focus on scientific solutions to the crisis. The level of other users' engagement is the lowest across the clusters (both in terms of mean and median), and the proportion of negative replies is among the lowest. There is thus less interest from the public in this cluster than in the other clusters. For instance, the tweet written by a research centre that triggered the highest number of engagements promotes the elaboration of a tool to track COVID-19 cases.

In Cluster 5, the communication of health experts is the most salient and focuses on the testing policy. Tweets from this cluster essentially relate to the second normalization period as well as the third COVID-19 wave. This cluster triggers the highest median number of engagements from other users. The tweet triggering the highest number of engagements was written by the FOPH and announces the lifting of certain restrictions from July 2020. This tweet receives 111 replies, of which 35% are negatively loaded.

With respect to the target populations, which serve as a supplementary variable, we observe that the mentions of patients and elderly individuals are associated with the vaccination and certificate topics (see cluster 1). This is in line with the fact that the vaccination campaign started with older and more vulnerable sections of society. However, the communication of political and health authorities on Twitter is little focused on target populations, as the majority of tweets do not mention any target population.

## 5 Discussion and implications

The objective of this study was to compare offline and online trends to provide empirical evidence regarding how trust in health experts and political authorities has evolved throughout the different phases of the COVID-19 pandemic. This study contributes to understanding these processes by drawing on the Swiss context.

Our data collection shows interesting patterns. For instance, COVID-19 tweets emitted by actors replicated the COVID-19 case curve, including a lower level of Twitter activity between waves of the pandemic. This finding is in line with the results obtained by Pang et al. (2021), which showed that government engagement on social media was relatively low at the beginning of the pandemic and then surged in the acute stages, with a trend towards a decrease in engagement during the chronic stages. Federal institutions tweeted less about COVID-19 than health experts did due to the much broader range of topics that political authorities have to deal with. As such, political authorities tweeted significantly more about non-COVID-19 topics than health experts did. Overall, the COVID-19 tweets of experts triggered more engagement and replies from other users than those of authority figures (e.g., Federal Council executives). This is in line with findings that experts are more likely to be listened to than political authorities (Drescher et al., 2021).

Our first research question asked whether the level of public trust measured through opinion surveys is reflected in other users' engagement and negativity in replies. Comparing negativity in replies with trends in public trust from opinion surveys shows a similar decrease in public support for experts. Furthermore, the general decline in the mean rate of engagement from other users suggests that while the initial COVID-19 outbreak was characterized by increased trust in scientists and health authority experts, there was weakened trust in public health authorities as exposure to the epidemic became prolonged (Battiston et al., 2021). Overall, we found little congruence between the survey measure of trust and social media trends in terms of engagement and replies. This lack of congruence might suggest that people express more dissent on the internet than in surveys because either surveys are biased by desirability effects or the reply feature in Twitter is used mostly by discontented people, as tweets are unsolicited reactions. Either way, the findings reveal a complementarity need between the two data sources.

Our second research question focused on the salient associations between the topics, the actors, and the different stages of the online COVID-19-related discussions. We found that health experts, research centres, and cantonal authorities contributed greatly to the formation of the correspondence space. Furthermore, these actors focused on distinct topics and target audiences. For instance, the cantonal authorities spent an essential part of their communication on broadcasting the COVID-19 case and promoting the vaccination (as well as the certificate). Thus, patients and elderly individuals constituted their target audiences. Health experts were oriented towards the promotion of the testing policy, while the research centres focused on technological innovations and tracing applications.

Our third research question asked how the extracted clusters were received by a broader audience. We confirmed that the cantons played an important role in the management of the pandemic, notably due to federalist and subsidiarity principles (e.g., by being in charge of broadcasting and the application of COVID-19 policy measures at the local level). The fact that the cluster characterizing cantonal communication triggered one of the lowest levels of negativity suggests that cantonal authorities were able to build a good online followership and reputation for managing the crisis. The close reading of emblematic tweets from the clusters in terms of engagement and negativity also enabled us to highlight the variety in other user supportive and contesting behaviours towards the major actors during the pandemic. As in other European countries and the rest of the world, we found evidence that Switzerland is experiencing a mobilization against COVID-19 policy measures.

### 5.1 Theoretical contributions

From a theoretical perspective, the results of this study provide helpful conclusions about the communication between government authorities and the population in (health) crisis situations. Most notably, it reveals that the type of actors, the type of content, and the stages of public debate have an impact on other users' reactions to actors' messages. This is particularly important because actors' tweets can reach a large audience, potentially helping to raise public awareness and public acceptance of policy measures whose reception has been, to date, measured mostly through surveys. Relying on social media data enables us to access unsolicited behavioural and rhetorical measures that can be compared to survey results. Our findings resonate with the study of Gilardi et al. (2021), which states that social media challenge the capacity of political and media elites to craft a consensus regarding the appropriateness of different measures as responses to a major crisis. Indeed, actors seem to have a limited capacity to influence broader audience opinion. In this study, we show a low correspondence between social media and survey data sources, but it may well be that in other countries, we could have observed more contesting behaviours on social media (e.g., Haupt et al., 2021). The methodology used in this paper could be applied from a cross-country perspective. We found some evidence of public fatigue in Twitter features. We therefore encourage future studies to link online reactions to offline attitudes to account for the role played by social media in the public debate and to assess whether online and offline opinions are congruent.

### 5.2 Implications for practice

The results of this study have implications for governments, health organizations and experts, the media, and researchers in selecting suitable communication strategies that may foster the active liking and retweeting of messages on social media. For instance, we found a general decrease in the number of engagements from other users to tweets from health and political experts. This might be due to the fatigue effect in the public, which, in turn, might increase public concern about the legitimacy of COVID-19 policy measures. A better understanding of the communication and content dynamics among authorities and (online) public debate is thus pivotal to ensure the well-functioning of democratic institutions. Furthermore, we show that tweets that are clearly linked to a policy issue tend to trigger more engagement (in terms of likes and replies) from other users. This suggests that actors should adopt a communication strategy that promotes and discusses clear policy recommendations and measures instead of adopting a broadcasting behaviour (e.g., tweeting about the number of COVID-19 cases). Moreover, there is an incentive for actors to make use of hashtags (instead of mentions or links) to generate public attention and approval.

## 6 Conclusion and outlook

The findings obtained in this study enable us to improve our understanding of how the types of actors emitting messages, the variety of COVID-19-related topics, and the stages of the public debate all affect the reception of the messages. Social media play an important role because they allow actors to bypass institutional gatekeepers – such as political parties and newspapers – with a view to achieving public compliance with the promoted policy measures and motivating citizens to adopt preventative measures.

Despite signs of rising fatigue characterizing the later stages of public debate, our results indicate that actors' efforts to communicate on social media are generally well received by the online audience. This overall positive picture reflects public support for governmental authorities, as demonstrated during the two popular votes about the modification of COVID-19 measures. The first law was supported by 60% of voters on June 13, and the second saw an increased share of public support, with 62% of voters on November 28.

Our study contributes to political communication research in times of crisis by investigating how actors' online messages resonate with and are received by the wider audience. To the extent that elite communication is crucial for compliance with policy measures, the findings suggest that social media may hamper success in achieving COVID-19 responses in the later stages of public debate, as there seems to be increasing fatigue among the public. It is difficult to provide officials with a clear pathway to communicate their crisis response through social media. However, we can formulate the following recommendations: messages should be oriented towards specific policy issue measures instead of merely broadcasting statistics, messages should make use of content features such as hashtags, and cantonal authorities should continue to play a decisive role in crisis communication.

Building on our methodology, future research could adopt a cross-country perspective to assess the extent to which our conclusions are generalizable to the context of other countries. Furthermore, other popularity measures could be used to assess actors' reputation, such as the evolution of a network of followers. Finally, Twitter is a particular social media platform that encompasses a specific audience (and is perhaps more elitist than Facebook), and it is possible that this could have impacted our results, as the actors may have been depicted more positively on Twitter than on other platforms.

## Contribution

Maud Reveilhac planned the study, conducted the data analysis, and wrote the manuscript.

## Funding

None.

## Conflicts of interest

We have no conflict of interests to disclose.

## Figures and Tables

**Table:** 

Phase	MOSAïCH waves	Label	Covid-19 wave	Explanation
January to March 2020		W0		Detection of the first cases and a rapid increase in the number of cases.
March to April 2020		W1	1st wave	Peak in the epidemic and the establishment of a state of emergency. At the end of March 2020, the Confederation appoints a scientific advisory board called the *Taskforce* to find the best approach to overcoming the pandemic. A first measure establishes a policy on testing, isolation, and quarantine.
end of April to mid-June 2020	1st wave (start)	N1		Decrease in the number of cases and a relaxation of measures. The Confederation demonstrates its interest in an official mobile contact tracking application *SwissCovid (*launch of the testing phase on May 25, and public availability of the application on June 25). The application had been downloaded by 2.1 million inhabitants, or 25% of the population, 6 weeks after its release.
mid-June to September 2020	1st wave (end)	N2		End of the state of emergency and a further increase in cases. A nationwide face-mask rules for public transportation is introduced on July 6th.
October to December 2020	2nd wave (start & end)	W2	2nd wave	Tracing is ensured in the face of the very big increase in cases during the second wave of infections in the fall of 2020. At the end of December 2020, Switzerland begins a national vaccination campaign against Covid-19.
January to May 2021	3rd wave (start & end)	W3	3rd wave	Teleworking becomes compulsory, and shops not selling everyday consumer goods are closed. The third wave takes place in April 2021. The relaxations will be phased in until the end of May 2021.
mid-May to August 2021		N4		Public spaces (e.g. restaurant's terraces, cinemas, theatres or sport stadiums) partially reopen. Wearing masks and collecting contact details remains compulsory.
September to mid-November 2021		W4	4th wave	The fourth wave takes place in September 2021. The Federal Council announces that the covid certificate will be mandatory in indoor restaurants and other public spaces.
mid-November to mid-December 2021		W5	5th wave	Switzerland observes the first case of the *Omicron* variant which also coincide with the beginning of the fifth pandemic wave.
mid-December 2021		N5		The “*2G*” rule (for *geimpft* and *genesen*, i.e. vaccinated or cured) becomes the norm on December 20 for visits to public establishments and the so-called “2G+” rule comes into force implying that the last dose of vaccine must have been given within the last four months.

**Table:** 

Groups of seed actors	List of accounts
***University research centres***	ETH_Rat, Conseil_EPF, ETH, EPFL, snf_ch, fns_ch, CH_universities, academies_ch, SAGW_CH, UniBasel, unibern, UniFreiburg, UNIGEnews, USI_university, unil, UniLuzern, UniNeuchatel, HSGStGallen, UZH_ch
***News media***	19h30RTS, 20min, 20minutesOnline, 24heuresch, 52minutesRTS, AargauerZeitung, Ageficom, AppenzellerZeit, arcinfo, BauernZeitung1, bazonline, BernerZeitung, bielertagblatt, bilanmagazine, blickamabend, Blickch, bodenseewoche, bote_online, CdT_Online, chmediaag, Der_Landbote, derbund, die_weltwoche, energy_ch, Forum_RTS, Friburgera, Gauchebdo, giorndelpopolo, gruyere_journal, GTGrenchen, gvaobserver, heidi_news, info_sept, JournalduJura, LaCoteJournal, laliberte, LaNotizia, laregione, LaRegionNV, LausanneCites, lecourrier, lemanbleutv, Lematinch, lematindimanche, lenouvelliste, letemps, Lillustre, LuzernerZeitung, LuzeRund, mag_bonasavoir, MigrosMagazin, migrosmagazine, miseaupoint, Mittellaendisch, News_Luzern, Nouvo, NZZ, NZZaS, OstschweizamSon, radio24, radio3i, radiortn, RadioTeleSuisse, RepublikMagazin, RSInews, RSIonline, RTSinfrarouge, RTSpresse, RTSredaction, RTSUnDeux, SchweizerBauer, schweizerillu, SHN_News, SoBlick, sonntagsblatt, sonntagszeitung, SRF, srf_ostschweiz, srfaarau, srfbasel, srfbern, srfdata, srfkultur, srfluzern, srfnews, srfzuerich, suedostschweiz, swissinfo, swissinfo_de, swissinfo_en, swissinfo_fr, swissinfo_it, SZSolothurn, tagesanzeiger, tdgch, TeleBaernTV, Teleticino, TeleZueri, tempsprésent, Ticino7_CH, ticinonews, Ticinonline, watson_news, Weltwoche, weltwocheonline, WillisauerBote, Wochenzeitung, ZSZonline, zt_info, zuerisee
***Business and industry***	foraus, Avenir_Suisse, Avenir_Suisse_f, Avenir_Suisse_i, SGE, gewerbeverband, BaumeisterCH, arbeitgeber_ch, economie_suisse, economiesuisse, GewerkschaftSGB, SyndicatUSS, TravailsuisseCH, usp, sbv, santesuisse, doctorfmh, spitexch, publichealth_ch, SwissBankingSBA, GastroSuisseCH, hs_politik
***Cantonal authorities***	CantonduJura, Etat_Neuchatel, EtatdeVaud, GE_chancellerie, Etat_Fribourg, cantondeberne, kanton_bern, KantonSolothurn, Kanton_BL, BaselStadt, kantonaargau, KantonLuzern, CantonduValais, Kanton_Obwalden, KantonNW, infokantonuri, KantonZug, KantonZuerich, Kanton_Thurgau, kantonsg, AppAusserrhoden, Kanton_GR, ti_SIC
***Swiss Parliament***	ParlCH
***Political parties***	BDPSchweiz, Mitte_Centre, evppev, FDP_Liberalen, GrueneCH, grunliberale, LEGAdeiTicinesi, LesVertsSuisses, GrueneCH, PBDSuisse, PLR_Suisse, PSSuisse, pst_pop, solidariteS_CH, spschweiz, SVPch, UDCch, vertliberaux
***Federal Council***	BR_Sprecher, alain_berset, Violapamherd, ignaziocassis, s_sommaruga, ParmelinG, EDA_DFAE, EDI_DFI, EJPD_DFJP_DFGP, vbs_ddps, efd_dff, DefrWbf, UVEK_DETEC
***Federal Office of Public Health***	BAG_OFSP_UFSP
***Task force***	SwissScience_TF, TanjaStadler_CH

**Table:** 

generic	Distancing	face mask	contact tracing	quarantine	vaccine	pass
.*covid.*	social distancing.*	atemschutz.*	covid-app.*	lockdown.*	geimpf.*	hygieneausweis.*
.*corona.*	social-distancing.*	ffp2	dp-3t	lock-down.*	impfstoff.*	pass sanitaire
.*cov19.*	distanciation sociale	hygienemaske	dp 3t	quarantaine.*	impfen	passanitaire
.*cov2019.*	distance sociale	maske.*	dp3t	quarantän.*	impffrei.*	passe sanitaire
.*sars-cov.*	soziale distanz.*	maskenpflicht.*	kontakt.*rückverfolg.*	stayhome.*	impf-frei.*	passsanitaire
.*sarscov.*	distanzregel.*	maskenwahn.*	kontakt.*verfolg.*	confinement.*	impfnebenwirkung.*	passe-sanitaire
.*ncov.*	Distanzierung	maskenzwang.*	swisscovid.*	confiner	impf-nebenwirkung.*	pass-sanitaire
.*n-cov.*	Distanciation	masque.*	contact.*tracing.*	confiné.*	impfpässe	sanitary pass
anticovid.*		masquer	tracing	lockerung.*	impfplicht.*	zertifikatspflicht
anti-covid.*		schutzmaske.*	traçage	isolement.*	impf-plicht.*	.*zertifikat.*
.*taskforce.*		masks	tracer	quarantine	impfquote.*	.*certificat.*
.*task-force.*			contact-tracing.*	isoliering	impfrückstand.*	.*passcovid.*
ausserordendliche lage			corona app		impf-rückstand.*	.*covidpass.*
besondere lage			coronaapp.*		impfung.*	.*covid-pass.*
situation extraordinaire			corona-app.*		impfzwang.*	.*pass-covid.*
crise sanitaire			coronawarn.*		impf-zwang.*	passe-covid.*
crisesanitaire			corona warn.*		moderna	passe covid.*
gesundheitskrise.*			corona-warn.*		pfizer	
.*cv19.*			coviddapp.*		.*vaccin.*	
.*infektion.*			covid-codes		verimpf.*	
infection.*			covid app.*		anticorps	
pandemie			kontakt-rückverfolg.*		anti-coprs	
pandémie			kontakt-verfolg.*		antikörper.*	
beatmungsgerät.*					.*imunität	
respirateur.*					immunité	
respirator.*						
epidemie						
epidémie						
hospitalis.*						

**Table:** 

Actor information			Covid-19 tweets			Popularity measures		
Actor groups	#accounts	#tweets		#Covid-19 tweets	#cleaned tweets in German or French	Mean reply	Mean retweet	Mean like
Business & industry	22	13472		2385 (18%)	2036	0,90	1,26	3,41
Cantonal authorities	23	28624		7647 (27%)	6405	1,46	2,61	6,64
Federal Council	13	15894		2735 (17%)	1946	13,08	13,57	43,99
FOPH	1	5115		3359 (66%)	2157	14,85	15,63	36,35
News media	91	596820		87746 (15%)	59126	1,49	1,48	3,82
Political parties	17	17005		1783 (10%)	1733	6,83	7,09	28,46
Politicians	152	57563		7208 (13%)	6195	6,55	7,28	44,46
Swiss Parliament	1	2102		338 (16%)	213	0,74	1,99	5,57
Taskforce	1	283		176 (62%)	80	6,56	10,52	25,93
Taskforce board	1	253		97 (38%)	30	3,33	20,30	58,28
University research centres	19	23160		2126 (9%)	1745	0,35	3,49	7,94
Total	189	760291		115600		5,10	7,75	24,08

**Figure:**
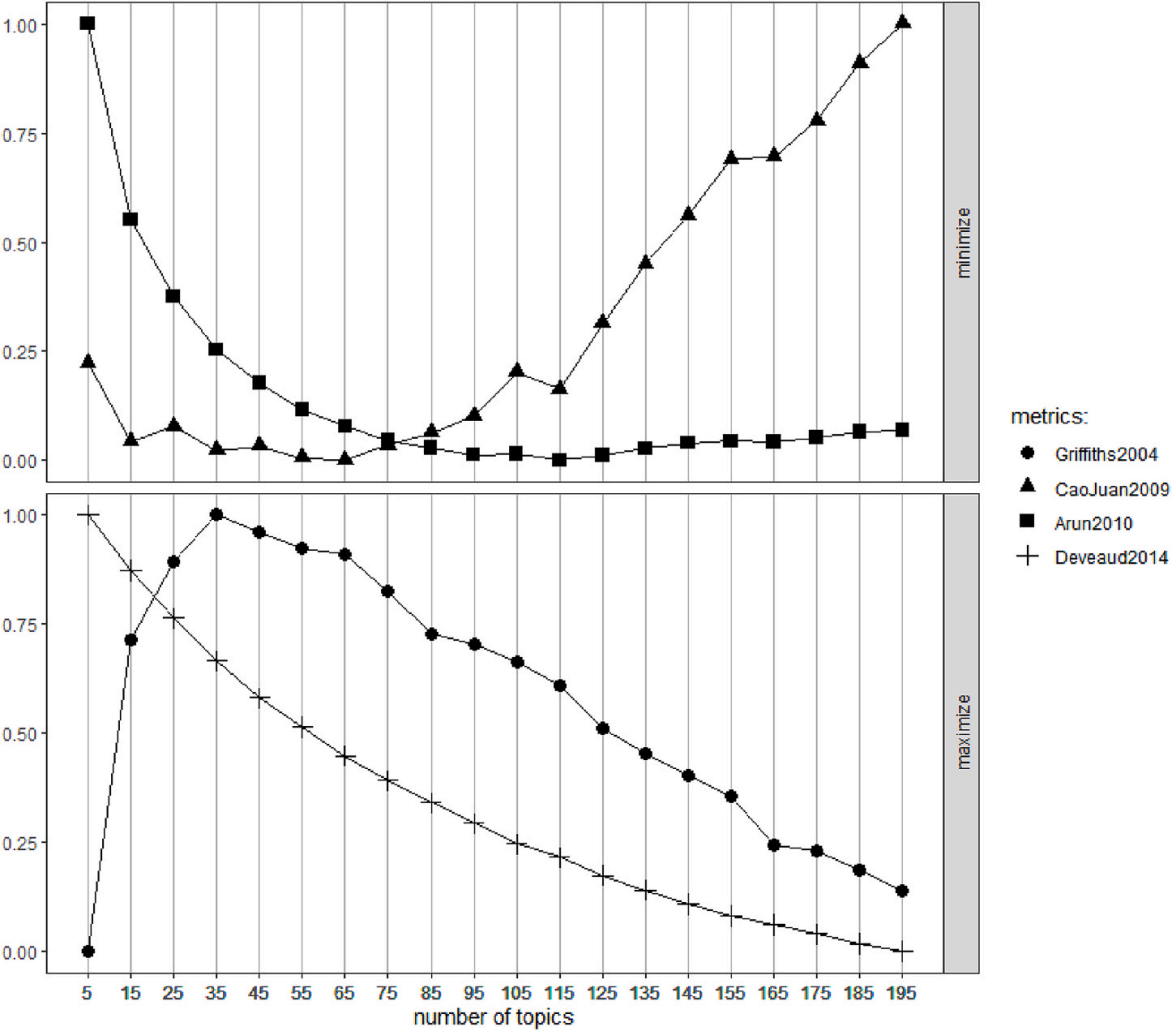


**Figure:**
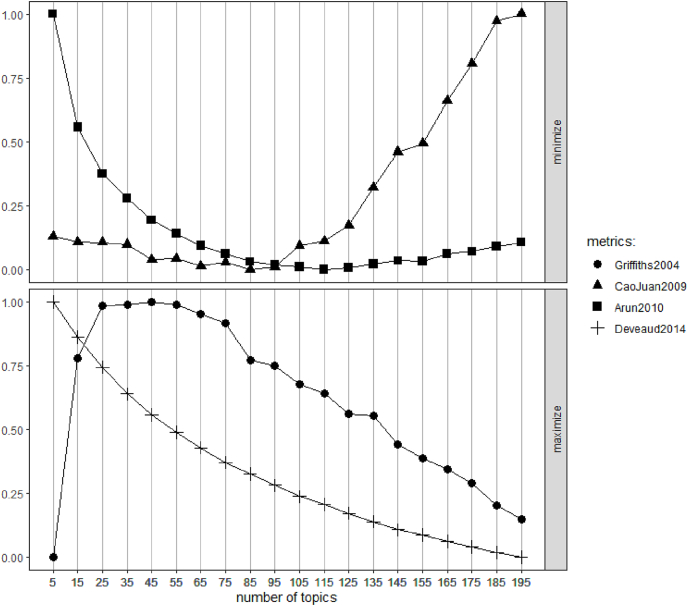


**Table:** 

language	children	Elderly	women	patients	adultes
French	enfant.*	personne.*âgée.*	femme.*	patients	adultes
	étudiant.*	personne.*agée.*	enceinte.*	hospitalisé.*	population
	bébé.*	retraité.*	mère.*		peuple
	école.*	maison.* de retraite	maman.*		
	écolier.*	EMS			
	écolière.*				
	gymnase.*				
	gymnasien.*				
German	kind	alte person.*	frau.*	patient.*	erwachsene.*
	kinder.*	ruhestand.*	schwanger.*	hospitalisierte.*	population.*
	baby	altersheim.*	mutter.*		Volk
	student.*	pflegeheim.*	mama.*		
	schüler.*	APH			
	schule.*				
	gymnasium				

**Figure:**
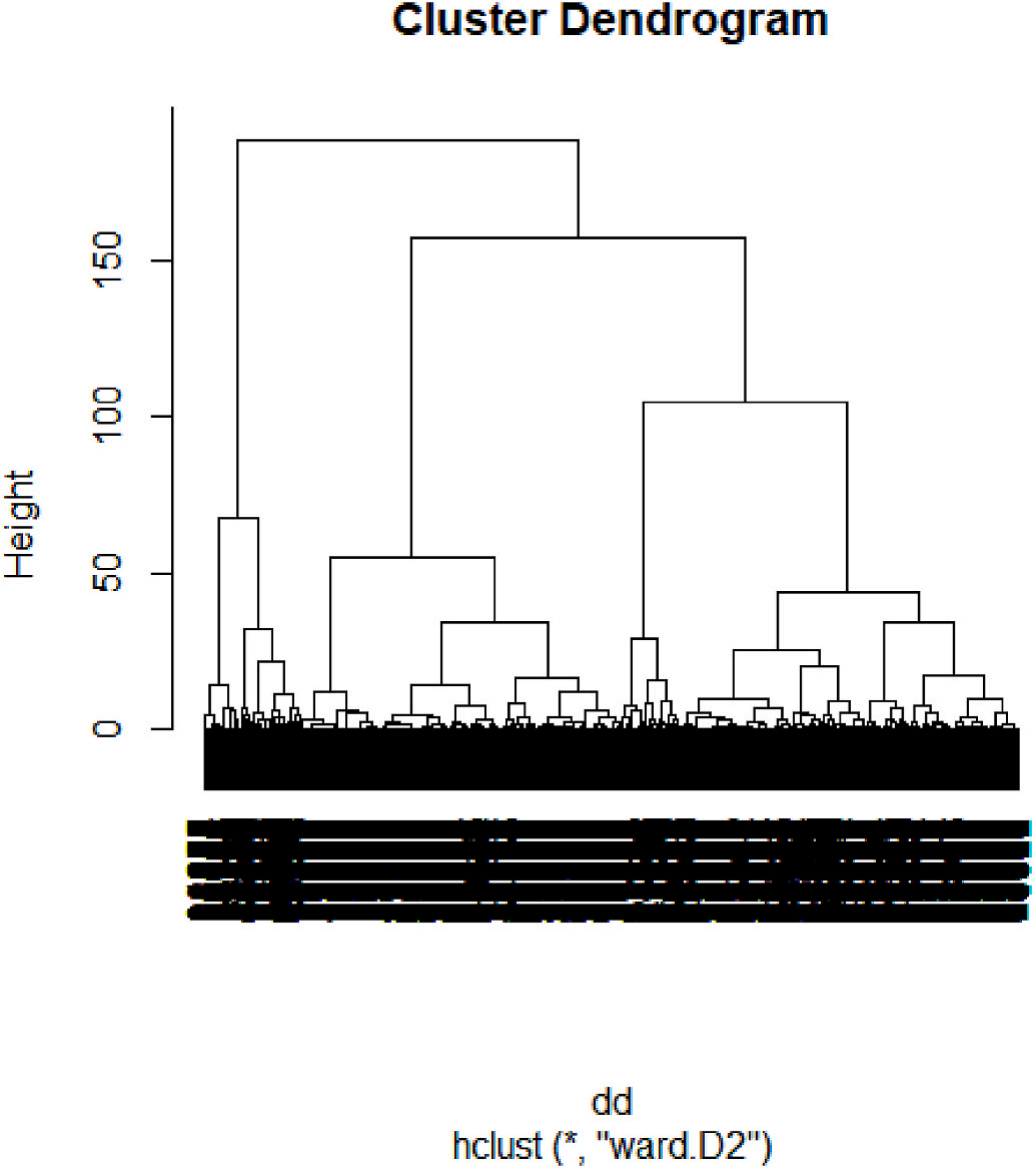


**Figure:**
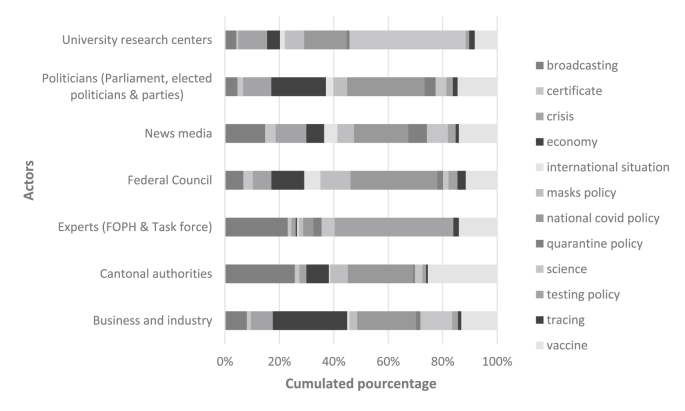


## Footnotes

1 For more information, please see: https://www.swissubase.ch/en/catalogue/studies/13871/16853/overview.
